# Application of omics technologies for a deeper insight into quali-quantitative production traits in broiler chickens: A review

**DOI:** 10.1186/s40104-018-0278-5

**Published:** 2018-09-10

**Authors:** Marco Zampiga, Joshua Flees, Adele Meluzzi, Sami Dridi, Federico Sirri

**Affiliations:** 1 Department of Agricultural and Food Sciences, Alma Mater Studiorum - University of Bologna, Via del Florio, 2, 40064 Ozzano dell’Emilia, Italy; 2 Center of Excellence for Poultry Science, University of Arkansas, Fayetteville, AR 72701 USA

**Keywords:** Broiler chicken, Feed efficiency, Meat quality, Nutrition, Omics technologies

## Abstract

The poultry industry is continuously facing substantial and different challenges such as the increasing cost of feed ingredients, the European Union’s ban of antibiotic as growth promoters, the antimicrobial resistance and the high incidence of muscle myopathies and breast meat abnormalities. In the last decade, there has been an extraordinary development of many genomic techniques able to describe global variation of genes, proteins and metabolites expression level. Proper application of these cutting-edge omics technologies (mainly transcriptomics, proteomics and metabolomics) paves the possibility to understand much useful information about the biological processes and pathways behind different complex traits of chickens. The current review aimed to highlight some important knowledge achieved through the application of omics technologies and proteo-genomics data in the field of feed efficiency, nutrition, meat quality and disease resistance in broiler chickens.

## Background

In the last decade, the extraordinary results obtained by the animal genome sequencing allowed the development of several analytical techniques able to describe the global variation of genes, proteins and metabolites expression level. Whereas genomic information remains constant during the lifespan of an animal, gene products such as proteins and metabolites change their expression levels in a rapid and dynamic manner, being regulated by a plethora of different environmental and physiological factors. Transcriptomics, proteomics and metabolomics are the main omics technologies currently used to investigate the expression profile of genes, proteins, and metabolites, respectively. The detailed description of these analytical techniques is beyond the scope of this paper and therefore they will be mentioned shortly. Briefly, transcriptomic aims to identify the expression levels of genes in mRNA transcripts in response to different environmental stimuli or during specific patho-physiological conditions, as well as to identify genes underlying specific traits. Northern blotting, real-time quantitative reverse transcription PCR (RT-PCR), microarray and RNA-sequencing (RNA-seq) are the main analytical platforms currently applied in transcriptomics studies. However, it’s known that mRNA levels in a cell do not really reflect those of the corresponding protein. Therefore, it might be useful to study the proteome, which is defined as the global set of proteins and all their post-translation modifications expressed in a cell/tissue/organ at a given time during specific conditions [1, 2]. Due to the wide differences in chemical and physical properties of proteins, and because no amplification method is provided for them, proteomic studies mainly rely on several chromatographic and electrophoretic methods to separate proteins [3], which can be subsequently identified using mass spectrometry (MS) combining soft-ionization techniques with different mass analyzer [4]. Other analytical techniques available for proteins are nuclear magnetic resonance (NMR) or immunological methods such as Western blot. Finally, metabolomics represents the quanti-qualitative study of a wide range of small biological metabolites [5], either deriving from the genome expression (endogenous metabolites) or not (e.g., xenobiotic metabolites, such as environmental pollutants or drugs) [6]. Usually, different biological samples can be analyzed through NMR or MS approaches in order to identify metabolites showing differential expression in relation to different conditions or stimuli (e.g., diseases or dietary treatments) or to discover biomarkers useful to discriminate animals or animal products with different characteristics [7]. Although each of the above mentioned analytical platforms provides very useful outputs, they are only able to describe “a part of the entire biological picture” if considered singularly. Liebler [1] reported that each protein, regardless its role and form, expresses a function that assumes significance only in the context of all the other functions and activities also being expressed in the same cell. Therefore, the next step is to integrate all the information obtained by the different omics platforms using appropriate bioinformatics and statistical tools. This relatively new approach, called system biology, provides a holistic and methodological overview of the entire biological system rather than its singular components alone [8].

## Feed efficiency

**Fig. 1:**
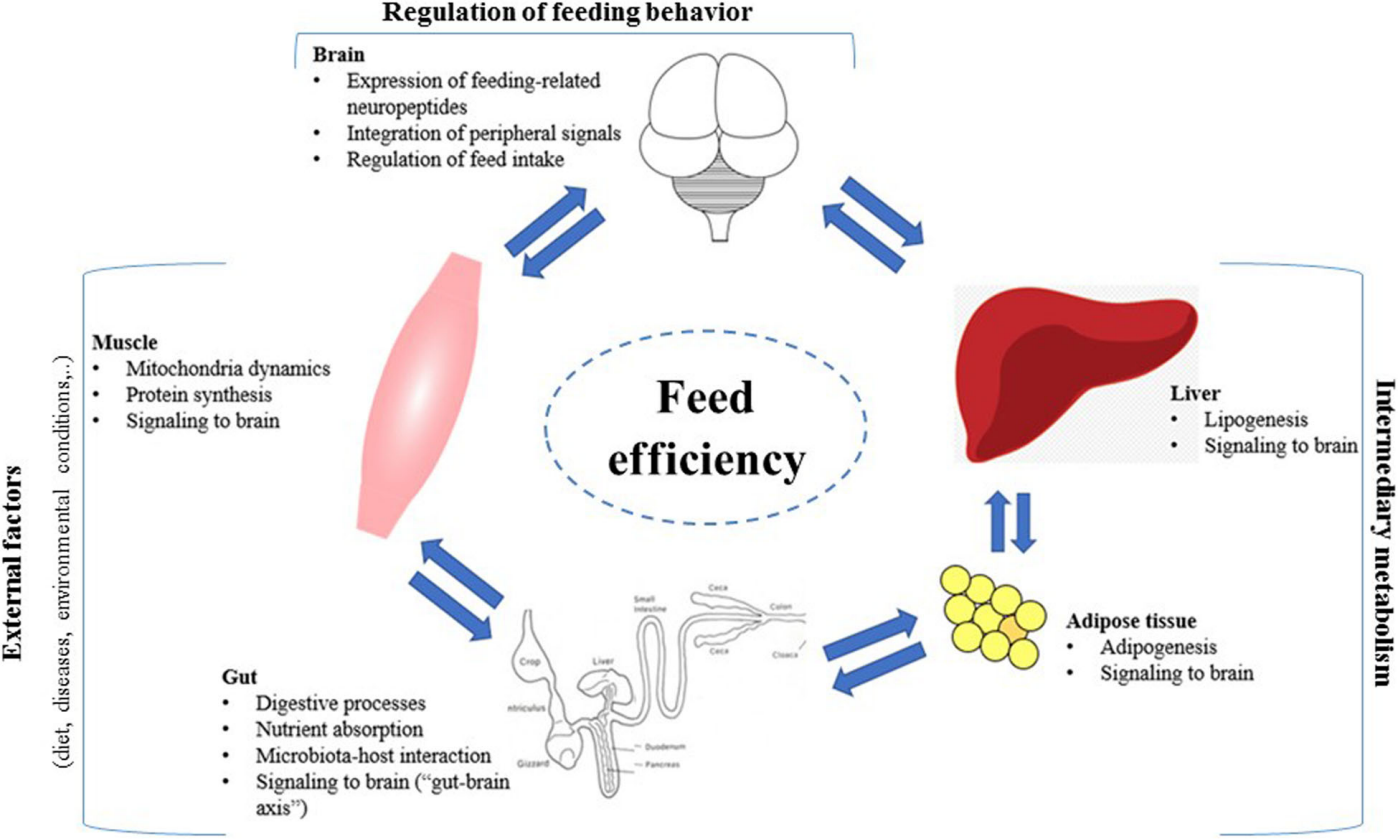
Overview of the physiological factors involved in the determinism of feed efficiency in broiler chickens

Feed efficiency (FE) represents one of the most important and complex traits in livestock production since up to 70% of total production costs is given by feed. FE could be defined as the ability of an animal to convert feed into body mass. In animals, FE could be assed using different parameters such as feed conversion rate (FCR), which represents the ratio between feed intake and body weight gain for a specific period of growth, or residual feed intake (RFI), which is the variation between actual and expected feed intake of an animal based on the estimated requirement for its maintenance and growth/production [9]. Therefore, FE could be considered as the net result among feed consumption, which is determined by the voluntary feed intake and its regulatory mechanisms, and energy expenditure, which is affected by the maintenance metabolism, the rate of anabolic processes and the intermediary metabolism in different tissues and organs (Fig. 1). Many efforts were conducted to date to understand the molecular aspects in different tissues of broiler chickens which may exert a huge effect on the overall expression of FE phenotype.

## Intermediary metabolism

### Muscle

#### Mitochondria dynamics and bioenergetics processes

In chickens, muscle is the main site for thermogenesis since they lack the brown adipose tissue. Being one of the main metabolic organs, the bioenergetics processes within the muscle can deeply influence FE in broilers. As mitochondria are responsible for producing around 90% of the energy pool for cells, studies have been conducted to evaluate whether the expression of different FE phenotypes would be associated with differences or inefficiencies in muscle mitochondria structure and functionality. The first confirmation of this hypothesis was obtained by Bottje et al. [10] when a potential link between muscle mitochondria functionality and the phenotypic expression of FE was established in a broiler breeder line. The birds, belonging to the same genetic line, were held in thermoneutral environment in individual cages, fed the same diet and individually phenotyped for FE, and therefore any behavioral, environmental or dietary effect was excluded from the FE equation [11]. At the gene level, differences in the expression of genes involved in mitochondria biogenesis [peroxisome proliferator-activated receptor-γ (*PPAR-γ*), PPAR-γ coactivator-1α (*PGC-1α*) and inducible nitric oxide synthase (*iNOS*)] and energy metabolism [avian adenine nucleotide translocator (*avANT*), cytochrome oxidase III (*COX III*), and avian uncoupling protein (*avUPC*)] were observed in breast muscle of birds showing either high or low FE [12]. Regarding the physiological aspects, the activity of mitochondria complexes I, II, III, and IV has been reported to be higher in breast muscle of high FE birds compared to low ones [13]. Previously, Bottje et al. [11] reported that the activity of complex I and II was greater in breast and leg mitochondria of high FE birds. Recently, the upregulation of genes associated with electron transport chain (ETC) complex I [14], as well as the greater predicted activity of complex I, III, IV and V [15] in breast muscle of high FE birds, seem to confirm an overall increased activity of mitochondrial complexes in the high FE phenotype. To address whether these differences in respiratory chain complexes activity might be due to an altered expression of mitochondria proteins, post-translational modifications or oxidative damages, different proteo-genomics approaches were performed. At the protein level, mitochondrial ETC complexes should not be considered as single entities but rather the assemblies of multiprotein subunits, which expression is controlled by both nuclear and mitochondrial DNA [13]. Although the activity of the different complexes appeared higher in most of the reported studies, no significant differences have been observed in complex I protein expression, as well as in the expression of 70S subunit of complex II or α-ATPase (complex V) in breast muscle of birds with different FE phenotype [13]. Nonetheless, cytochrome b, cytochrome c1, core I (complex III) and cytochrome c oxidase subunit II (complex IV) showed higher expression in low FE mitochondria than in the high FE ones [13]. Considering other chicken tissues over than muscle, only two mitochondrial proteins (cytochrome c1 and cytochrome c oxidase subunit II) exhibited differential expression between high or low FE birds in at least 4 out of 5 examined tissues (breast muscle, heart, duodenum, liver, and lymphocytes), suggesting the existence of tissue-specific regulatory mechanisms (e.g., post-translational modifications or different cell turnover) [13, 16–19]. On the other hand, Kong et al. [15], using a shotgun proteomic approach, showed a higher mitochondria proteins expression in breast muscle of high FE birds belonging to the same broiler breeder line and identified “mitochondrial function” and “oxidative phosphorylation” as first and fifth top expressed pathways, respectively. Moreover, it has been reported that the activation of upstream regulators such as progesterone and triiodothyronine would be associated with the increased expression of mitochondria proteins in the high FE phenotype [15]. A common feature among the previously mentioned studies was the significantly higher level of oxidized mitochondria proteins in the tissues of low FE chickens, as indicated by the increased amount of protein carbonyls. Therefore, as suggested by Bottje et al. [20], the lower respiratory complex activity observed in low FE mitochondria might be due to the increased level of oxidized proteins rather than a reduced expression of ETC protein subunits. In conclusion, from a physiological perspective, Bottje and Kong [11] indicated that at least 2 physiological processes would have contributed to mitochondrial inefficiency and hence to the overall expression of a low FE phenotype. The first physiological process was site-specific defects in ETC that may have increased reactive oxygen species (ROS) production. In turn, the higher levels of ROS were identified to be responsible for the greater amount oxidized proteins observed in the low FE phenotype [20, 21]. An increased oxidation of mitochondrial proteins might have played a detrimental effect on FE since energy might have been directed towards reparation and synthesis of mitochondria proteins rather than for anabolic processes [11]. The second process associated with inefficiency was proton leak, which is a movement of protons across the inner mitochondrial membrane at other sites rather than through ATP synthase. Proton leak is fundamental for maintaining homeostasis by reducing mitochondrial ROS production, even though it represents an energetic wasteful process and accounts up to 50% of basal oxygen consumption rate in mitochondria [11]. Bottje et al. [22] reported that proton leak rates in the low FE phenotype were higher, or at least similar, to those observed in the high FE one. Finally, ROS, acting as secondary messengers, may have influenced the expression of genes and proteins involved in mitochondria functionality, activity or development [11].

#### Protein synthesis and cellular anabolic processes

On the same broiler breeder line, breast muscle global mRNA expression was assessed using a microarray-based approach [14, 23]. High FE birds were characterized by an upregulation of genes either involved in anabolic processes (protein packaging and scaffolding activity, purine and pyrimidine biosynthesis, prevention or delay of apoptosis and modulation of gene transcription), or related to major signal transduction and cascade mechanisms pathways [Protein kinase-A (PKA), c-Jun NH(2)-terminal protein kinase (Jnk), retinoic acid and retinoid X receptor (RAR-RXR)] or in sensing the energy status and regulating energy production in the cell [Adenosine monophosphate AMP-activated protein kinase (AMPK) and protein kinase AMP-activated non-catalytic subunit gamma 2 (PRKAγ2)]. At the same time, high FE birds showed downregulation of genes associated with cytoskeletal organization, as well as cyto-architecture and integrity-related genes, major histocompatibility complex cell recognition, stress-related heat shock proteins and several platelet derived growth factors genes. A global overview of the cellular processes which might have contributed to the phenotypic expression of FE has been summarized by Bottje and Kong [11]. Recent findings also suggested a potential role of insulin receptor, insulin-like growth factor 1 receptor, nuclear factor erythroid 2-like 2 [15], progesterone [15, 24], as well as mechanistic target of rapamycin (mTOR) and protein degradation pathways [25], in the phenotypic expression of FE in broiler chickens. On the other hand, rapamycin independent companion of target of rapamycin (RICTOR), mitogen-activated protein kinase 4 (MAP4K4), and serum response factor were predicted to be downregulated in muscle of high FE chickens [15]. Combining gene and protein expression analysis, Bottje et al. [26] reported also an enhanced mitochondrial and cytosolic ribosomal construction, protein translation, proteasomes and autophagy, in breast muscle of high FE birds. On the other hand, consistently with previous findings, Kong et al. [15] highlighted that several upstream regulators involved in the activation of cyto-architecture-related genes were inhibited in the high FE phenotype. Overall, broiler chickens showing a high FE phenotype seem to achieve a greater efficiency through reducing the energy expenditure for maintaining cytoskeletal architecture and function, as well as for substituting damaged proteins, while directing energy towards anabolic-related processes that may enhance overall cellular efficiency [15]. However, considering the less organized cytoskeletal architecture observed in high FE birds, it would be interesting to evaluate whether the selective pressure applied to improve FE may have negatively contributed to the increased incidence and severity of muscle myopathies recently observed in fast-growing broiler genotypes.

Recently, the biological basis of the differences between high and low FE chickens was investigated by Zhou et al. [27] through mRNA-seq and pathways analysis. Despite previously reported studies, which were focused on a broiler breeder line, the research of Zhou et al. [27] was carried out on breast muscle of male chickens obtained by crossing three commercial pure lines. The RNA-seq analysis identified a total of 1,059 differentially expressed genes between high and low FE chickens. High FE birds had a greater expression of genes related to muscle development, hypertrophy, and remodeling, as well as a decreased expression of protein degradation and atrophy-related genes. Moreover, transcriptional factors involved in muscle development resulted upregulated in these birds. These results, associated with the predicted activation of growth hormone and insulin-like growth factor-I/phosphatidylinositol 3-kinase/protein kinase B (IGFs/PI3K/Akt) signaling pathways, might explain the higher breast yield observed in high FE birds. Other important findings were the upregulation of genes related to inflammatory response and macrophage infiltration, as well as an increased expression of glutathione s-transferase superfamily genes which encode for antioxidant proteins. Moreover, the activation of hypoxia-inducible transcription factor-1α seems to suggest that a hypoxic condition may occur in breast muscle of high FE birds, which has been ascribed either to the increased inflammatory condition, to the excessive muscle remodeling or to the higher production of ROS [27]. It is interesting to note that most of the biological features observed in breasts of high FE birds can overlap those observed by Zhou et al. [28] in breast muscle of birds affected by wooden breast defect. Even though Zhou et al. [27] reported no clinical symptoms of sickness or muscle damage, the similarity in gene expression profile may indicate common biological patterns and hence a possible relationship between FE and wooden breast incidence.

### Adipose tissue and liver

Adipose tissue plays a central role in energy homeostasis being a metabolically active organ with endocrine and regulatory functions. On the same chicken population of Zhou et al. [27], another RNA-seq analysis was conducted to investigate the gene expression profile in abdominal fat [28]. Low FE chickens showed higher lipid accumulation, which was likely determined by the upregulation of genes involved in lipid synthesis, as well as downregulation of genes enhancing triglyceride hydrolysis and cholesterol transport from adipose tissue. Moreover, the predicted activation of sterol regulatory element binding proteins, as well as the inhibition of insulin-induced gene 1, was consistent with the higher cholesterol accumulation observed in low FE birds [28].

On the other hand, adipose tissue has also a secretory function. Leptin, for instance, is a peptide hormone secreted by the adipose tissue which is involved in the regulation of feed intake and energy metabolism in both mammals and avian species. In chickens, leptin is expressed also in liver and it is regulated by the nutritional state of the birds [29]. As in mammals, leptin is recognized as “satiety hormone” in chickens as well, since it reduces feed intake and increases energy expenditure through the interaction with its receptor localized both in brain neurons and in other peripheral tissues [30]. Understanding the role of different molecules such as leptin in both central and peripheral tissues of the chickens is fundamental to increase our knowledge regarding the molecular basis of FE.

### Gut

The gut is one of the most important tissues able to influence the expression of different FE phenotypes due to its function in nutrient digestion and absorption, as well as for its immunological role (which will be discussed in the “Disease resistance” section) [31].

Ojano-Dirain et al. [12] found a higher level of oxidized proteins in duodenal mucosa homogenate and duodenal mitochondria of low FE birds. On the other hand, higher mRNA expression of *PPAR-γ* and *iNOS* was observed in the duodenum of high FE birds, whereas no significant difference was reported for *PGC-1α*, *avANT* and *COXIII* [12]. Lee et al. [32] analyzed the transcriptomic profile in duodenum of chickens divergently selected for RFI. The Authors observed that the selection process improved FE by reducing feed intake without significant changes in body weight gain. The molecular mechanism behind this improvement has been associated with the upregulation of genes involved in the reduction of appetite and increased cellular oxidative stress, prolonged cell cycle, DNA damage and apoptosis, as well as greater oxidation of dietary fats and efficient fatty acids transport from the intestine. Moreover, differential expression of genes involved in the avian target of rapamycin (avTOR) signaling pathways has been observed in liver and small intestine of meat-type chickens divergently selected for RFI, confirming a potential involvement of avTOR/PI3K pathway in determining FE in chickens [33].

Recently, the development of new omics technologies and platforms has strengthened the possibility to investigate the gut microbiota and its metabolic activities in farm animals [34]. Several papers reported differences in the intestinal microbiota between chickens showing different FCR [35–37]. Stanley et al. [35] observed no significant difference in jejunum microbiota composition between birds with high or low FCR as this tract was almost exclusively populated by members of the genus *Lactobacillus*. On the contrary, caecum microbiota showed higher diversity and 24 unclassified bacterial species were found to be differentially expressed between high and low performing birds. In a recent study, three families, Lachnospiraceae, Ruminococcaceae, and Erysipelotrichaceae, have been associated with the phenotypic expression of high FE [36]. In these birds, higher abundance of *Ruminococcus*, *Faecalibacterium*, *Clostridium* and two unknown genera from the Lachnospiraceae family was also observed. Even if some strains of *Lactobacillus* are aimed to improve broilers performance, Stanley et al. [36] identified others which have an undesirable outcome on the overall performance mainly through a stimulation of feed consumption. In another study, Mignon-Grasteau et al. [37] reported that birds selected for a low FCR showed lower cecal counts of *Lactobacillus*, *L. salivarius* and *E. coli* compared to the high ones. These variations in bacterial groups affected also the equilibrium between bacteria in the gut. Indeed, low FCR birds exhibited less *L. salivarius* and more *L. crispatus* to *Lactobacillus* ratio, as well as a higher ratio of clostridia to *Lactobacillus* and to *E. coli*. Albeit it has been calculated on a limited number of animals, the genetic heritability of microbiota was rather low, even if an appreciable heritability coefficient (between 0.16 and 0.24) was observed for the ratios of *L. crispatus*, *C. leptum* and *C. coccoides* to *E. coli*. Finally, the authors identified 14 quantitative trait loci (QTL) which can affect the composition of the microbiota, even if they resulted significant only on a chromosome-wide scale. Interestingly, the only QTL close to genome-wide significance (QTL for *C. leptum* on chromosome 6) was located in a region containing genes involved in inflammatory response and intestinal motility [37]. However, as emerged in three different trials performed by Stanley et al. [36], the microbiota associated with the phenotypic expression of FE resulted characterized by a great variability, indicating that other efforts should be done to identify probiotic bacteria and microbiota composition able to provide positive effects on FE.

### Brain

Feeding behavior and body energy homeostasis are intimately connected with the brain [30, 38–40], in particular with the infundibular nucleus of the hypothalamus [40]. Here, the hypothalamic melanocortin system contains two different populations of neurons which can modulate feed intake through the secretion of various neuropeptides. Briefly, a reduction of feed consumption is mediated by α-melanocyte stimulating hormone, released by proopiomelanocortin neurons, and cocaine- and amphetamine-regulated transcript. On the other hand, neuropeptide Y and agouti-related protein can stimulate appetite and increase feed intake by repressing the melanocortin anorexigenic effect [40]. Differences in the expression levels of these neuropeptides and some feeding-related genes have been reported in the hypothalamus of chickens [41] and quails [42] divergently selected for RFI and FE, respectively. Besides the brain, the mRNA expression of neuropeptide Y and its receptors has been detected in many peripheral tissues of the chicken, suggesting a crucial role of this neuropeptide in energy homeostasis processes [43]. However, other factors such as leptin [29] and several gut hormones [44] can affect central feed intake regulation and hence energy homeostasis in chickens.

Overall, FE appears as a very complex trait regulated by many different factors. Probably, the entire biological mechanism involved in the phenotypic expression of FE might be never understood, but increasing our knowledge on this trait might be useful to develop more efficient broilers through marker-assisted selection with undoubtedly positive effects on the economic and environmental sustainability of the poultry industry. The selective process aimed at improving FE in broilers has been extraordinary and has determined a reduction of FCR of approximately 50% in 50 years [45]. However, these improvements are coupled with some negative outcomes such as a hyperphagic feeding behavior and an increased proneness to obesity and muscle abnormalities, which will be discussed in detail in the next chapter.

## Meat quality

### Breast meat abnormalities

World population is continuously increasing, and the demand for poultry meat products is growing in the same fashion. The attractiveness of poultry meat is ascribable to its healthy profile (high protein, low fat, balanced *n*-6 to *n*-3 PUFA ratio, low levels of sodium and cholesterol), its relatively low price, and the absence of religious limitations related to its consumption. In the past years, the genetic selection has been strongly directed to increase breast muscle yield since breast meat represents the most appreciated and valuable part of the carcass. However, the marked improvements obtained in breast meat yield in fast-growing broilers has been coupled with a tremendous increase in the incidence of muscle abnormalities such as PSE-like, Oregon disease, white striping (WS), wooden breast (WB) and spaghetti meat defect (SM) (for review see [46, 47]). These myopathies lead to a reduction of technological, nutritional and sensorial traits of breast meat causing huge economic losses to the poultry industry. To date, there is no consensus around the etiology of these muscle abnormalities, even though the intense selection for high breast yield and growth rate seems to have played a fundamental role. Alnahhas et al. [48] reported a high heritability for WS defect, as well as a good correlation between its incidence and both breast meat and *pectoralis major* yield, indicating that a strong genetic basis could be defined for this myopathy. On the contrary, Bailey et al. [49] suggested that environmental and managing factors might be involved in the onset of this condition since they observed low values of heritability and a low genetic correlation with breast yield and body weight at slaughter.

From a nutritional point of view, breasts affected by WS showed a significant reduction of myofibrillar and sarcoplasmic protein content and solubility. Indeed, it has been identified a lower concentration of 3 myofibrillar proteins (actin; slow-twitch light chain myosin; and fast-twitch light chain myosin) as well as a reduction of almost all the sarcoplasmic proteins when WS occurs [50]. Breasts affected by both WS and WB showed a lower relative abundance of slow-twitch light chain myosin and greater amount of a 70 kDa myosin heavy chain fragment [50]. Also Vignale et al. [51] observed an increased degradation of muscular proteins in breasts with severe WS, as indicated by the higher fractional breakdown rate of muscle proteins and the upregulation of genes related to proteolysis in affected breasts [51].

The genomic transcription of breasts showing both WS and WB defects was investigated by Zambonelli et al. [52]. Microarray analysis identified 207 genes showing differential expression between affected and unaffected breasts. The former exhibited significant alterations in the expression level of genes related to muscle development, polysaccharide metabolic processes, glucose metabolism, proteoglycans synthesis, inflammation, oxidative stress and calcium signaling pathway. Overall, these results overlap those reported by Mutryn et al. [53] who performed a mRNA-seq analysis to identify differentially expressed genes and pathways associated to WB. Consistently with Zambonelli et al. [52], mRNA-seq analysis highlighted significant differences in genes involved in intracellular calcium level, oxidative stress, localized hypoxia, possible fiber-type switching and cellular repairing. As previously reported, some biological changes occurred in breasts showing WB abnormality are similar to those reported in breasts of high feed efficiency birds by Zhou et al. [27]. Therefore, according to the authors [27], it might be possible that the selection for increased feed efficiency and growth rate might have changed gene expression and molecular pathways in breast muscle, resulting in an increased incidence and severity of muscle abnormalities such as WB and WS.

Using a proteomic approach, a total of 141 differentially expressed proteins were identified between breasts with no or limited myopathic lesions and those showing severe muscle degeneration [54]. These proteins were mainly associated with cellular movement, carbohydrate metabolism, protein synthesis, post-translational modification and protein folding. Up-regulation of eukaryotic initiation factor 2 (eIF2) and 4 (eIF4), mTOR, and 70 kDa ribosomal S6 kinase (p70S6K) signaling may indicate an increased protein synthesis in degenerated breasts, which might be associated either with the ongoing regenerative processes or to the enhanced growth rate of breast muscle in these birds [54]. Glycolysis and gluconeogenesis were the main biological pathways predicted to be down-regulated in breasts with severe muscle degeneration [54]. On the other hand, breasts affected by both WS and WB showed a higher relative abundance of lactate dehydrogenase, glyceraldehyde dehydrogenase, aldolase, and glycogen phosphorylase, suggesting an enhanced glycolytic activity [52]. Recently, the results of the proteomic analysis performed by Cai et al. [55] seem to confirm the differences in the relative abundance of glycolytic proteins and oxidative stress conditions between affected and unaffected breast.

Considering metabolites, birds affected by WS showed no significant difference in hematologic profile, including leukocyte count, as well as in serum metabolites and electrolytes [56]. However, a significant increase in serum concentration of several enzymes related to muscle damage, such as alanine aminotransferase, alkaline phosphatase, aspartate aminotransferase, creatine kinase and lactate dehydrogenase, was observed when WS occurs [56]. Recently, an NMR approach allowed the detection of lower levels of anserine, carnosine and creatine in dystrophic chicken breast, indicating a possible alteration of muscle homeostasis and energy-generating pathways [57]. The metabolic profile of breast muscle affected by WB myopathy was investigated also using different MS approaches [58]. The identified compounds resulted mainly associated with an augmented oxidative stress, elevated protein levels, muscle degradation, and altered glucose metabolism. Interestingly, it has been reported an over-activation of the ascorbate biosynthesis pathway, which might be involved in glycogen depletion and oxidative stress in affected samples. Therefore, as pointed out by the authors [58], a potential beneficial role of dietary vitamin C supplementation in decreasing the incidence of WB myopathy can be hypothesized. Recently, also the expression of muscle-specific transcriptional regulatory factors, such as myogenic differentiation 1, myogenin, decorin, myostatin, and transforming growth factor beta 1, was reported to be significantly correlated with the overall phenotypic expression of WB [59]. However, the expression of these genes resulted not consistent between two different fast-growing chicken genotypes, suggesting that the etiology of the WB myopathy may vary among different commercial broiler lines [59]. Taken together, these results mainly suggest an alteration of carbohydrate metabolism and protein synthesis, as well as oxidative stress, localized inflammation and hypoxia, associated with the muscle abnormalities. In conclusion, even though the molecular mechanism behind muscle abnormalities need further insights to be better defined, the results obtained through the application of omics technologies allowed to understand important information which can be useful to limit the incidence and severity of breast muscle myopathies in broiler chickens.

### Meat quality attributes

Ultimate pH (pHu) is considered an important meat quality trait since it is strictly related to other attributes such as water holding capacity and color. Beauclercq et al. [60] characterized muscle and serum metabolites in chicken lines divergently selected for breast meat pHu (pH+ with higher pHu vs. pH– with a lower one) through high-resolution NMR. The selection process exerted significant changes in the metabolic profile of *pectoralis major* muscle and serum, as indicated by the discriminant analysis models in which a clear separation between the two chicken lines has been observed. The results of the metabolites set enrichment analysis, that was carried out to identify the metabolic pathways enriched in each broiler line, showed that the most representative metabolic pathways in the pHu− and pHu+ line were mainly involved in carbohydrate metabolism (e.g. glycolysis and gluconeogenesis), and amino acid and protein metabolism (e.g. phenylalanine−tyrosine metabolism and protein biosynthesis), respectively. As they stated, these results might indicate that the pH− line had a great ability to store glycogen in muscle and use carbohydrates as the main energy source, whereas the pHu+ one produced energy mainly through amino acid and protein catabolism, as well as lipid oxidation. Moreover, the authors identified a set of metabolites characterizing either the pHu+ or the pHu− line that, after a validation in an independent population of commercial broilers, might be used for molecular tests to predict breast meat quality and exclude from parental stock individuals that would present meat-quality defects [60].

Meat color represents the main visual factor affecting the consumer’s choice. In order to identify the gene underlying the chicken meat color QTL on *Gallus gallus* chromosome 11, Le Bihan-Duval et al. [61] merged a classical QTL analysis with gene expression QTL. The authors identified the beta-carotene monooxygenase 1 (*BCMO1*) gene, encoding for the β-carotene 15, 15′-monooxygenase, an enzyme responsible for the conversion of β-carotene into colorless retinal, as a good functional candidate. After gene sequencing, two fully-linked single nucleotide polymorphisms (SNP) were discovered within the promoter of the *BCMO1* gene and two haplotypes showing different promoter activity were defined. The two haplotypes exhibited significant differences in *BCMO1* gene expression and breast meat yellowness, consistently with the variation in lutein and zeaxanthin content observed in their breast [61, 62]. The genetic variant however did not impair growth performance and body composition of the chickens, as well as the expression of genes related to uptake and metabolism of carotenoids [62]. Moreover, the effect of the polymorphism on *BCMO1* gene expression and carotenoids concentration was not noticeable in other chicken tissues, such as liver, duodenum and *sartorius muscle*, indicating a tissue-specific effect [62]. Different proteogenomic approaches have also been applied to investigate the differences observed either in muscle growth or in meat quality traits among different chicken [63–65] and turkey [66, 67] lines. As well, genome-wide association studies have shown excellent results in identifying SNP and genes related to meat quality attributes [68–70]. In conclusion, omics technologies have exhibited an extraordinary potential to dissect and study some important topics concerning poultry meat quality. The results obtained through these analyses, as well as those deriving from the application of new approaches or the investigation of new traits, might be useful for both poultry producers and breeding companies in order to improve meat quality and carcass traits.

## Nutrition

### Efficacy of dietary treatments

Nutrition can be considered one of the most important environmental factors affecting genome expression. As well, nutrients should not be merely considered as a provider of nutritive principles but also a source of various molecules, which can be sensed by the organism and influence genome expression [71]. Therefore, a possible application of omics technologies in animal nutrition might be the identification of the molecular mechanism laying behind the phenotypic responses to the dietary administration of different kind of compounds and additives.

Considering macronutrients, dietary amino acids play a central role in protein metabolism (e.g. protein synthesis, proteolysis and amino acid oxidation). Besides this aspect, amino acids can also act as regulators of different metabolic pathways related to muscle development and mRNA translation into proteins [72]. It is well established that the dietary supplementation of lysine can improve growth performance and breast yield in broilers [73–75], but also meat quality traits such as water holding capacity and pH [75]. The dietary supplementation of lysine in lysine-deficients diet stimulated protein synthesis in skeletal muscle, whereas its dietary deprivation increased the fractional rate of protein breakdown (proteolysis) in *pectoralis major* muscle of growing chickens [76, 77]. Furthermore, also daily variations in dietary lysine content (sequential feeding) have been associated with an altered expression of genes related to proteolysis in breast muscle of chickens [78].

Methionine levels in the diet can deeply affect productive performance and breast meat yield in broilers [79–82]. It has been reported that dietary methionine altered the expression of myogenic genes (myogenic factor 5, myocyte enhancer factor 2B and myostatin) [81], as well as that of proteins mainly related to citrate cycle, calcium signaling, actin cytoskeleton and clathrin-mediated endocytosis signaling in chicken breast muscle [80]. A previous work showed that peptides belonging to three proteins (pyruvate kinase, myosin alkali light chain-1, and ribosomal-protein-L-29) were exclusively detected in breast muscle of chickens fed a methionine-deficient diet [79]. On the other hand, higher plasma concentration of uric acid and triglycerides was observed in response to the dietary supplementation of methionine [81]. Wen et al. [82] also reported that increasing the dietary methionine levels could be a valuable strategy to support productive performance and breast yield of chickens with a low hatching weight. As stated by the authors, these improvements were likely attributable to alterations in insulin-like growth factor-I synthesis and expression of genes involved in the target of rapamycin/eIF4E-binding protein 1 and forkhead box O4/atrogin-1 pathways [82].

Arginine is an essential amino acid for chickens and several studies have been conducted to evaluate the effects of its dietary supplementation on both productive and molecular aspects. Fouad et al. [83] reported that dietary arginine can modulate lipid metabolism as indicated by the reduced abdominal fat content, as well as the lower plasma triglyceride and total cholesterol concentrations in broilers fed arginine-supplemented diets. At the transcriptional level, arginine increased the expression of carnitine palmitoyl transferase1 (*CPT1*) and 3-hydroxyacyl-CoA dehydrogenase (*3HADH*) in the heart, while reduced that of fatty acid synthase (*FAS*) in the liver [83]. Arginine also showed positive effects on gut mucosa health and integrity in broilers subjected to coccidia challenge [84], as well as on attenuating the inflammatory response elicited by lipopolysaccharide treatment [85] and on the immunosuppression induced by infectious bursal disease virus challenge [86] (these papers will be discussed in detail in the “Immunomodulatory effects of nutrition” section).

Considering vitamins, Vignale et al. [87] observed that the dietary replacement of cholecalciferol (vitamin D_3_) with 25-hydroxycholecalciferol [25(OH)D_3_], a vitamin D metabolite available for commercial poultry use, increased breast meat yield and fractional synthesis rate of breast muscle proteins. Chickens fed 25(OH)D_3_ showed higher expression of vitamin D receptor, a DNA-binding transcription factor that mediates the action of vitamin D, and also a higher activation of the mTOR/S6 kinase pathway, highlighting the important role played by this pathway in mediating the effects of 25(OH)D_3_ on chicken muscle proliferation and development. These in-vivo results were corroborated by the in-vitro functional study performed on a quail myoblast cell line (QM7 cells) in which an increased expression of vitamin D receptor, as well as a greater translocation of it into cell nucleus, has been observed when cells were treated with 25(OH)D_3_. Nonetheless, 25(OH)D_3_ induced cell proliferation in a dose-dependent manner and its effect was suppressed by blocking the mTOR pathway with rapamycin [87]. Other interesting insights recently obtained in the field of broiler nutrition through the use of omics technologies regarded the effects of heat stress on gene expression and nutrients transporters in the jejunum [88], the evaluation of the dietary supplementation of branched-chain amino acids on the expression of hepatic fatty acids metabolism-related genes [89] and the modulation of intestinal phosphate transporters expression in response to phosphorous and phytase administration in the diet [90].

For what concern feed additives, the administration of phytase in broiler diet is reported to have a direct effect on organic phosphorus (phytate) and mineral digestibility, but also an indirect effect on zootechnical performance and muscle development mainly through the release of myo-inositol [91, 92]. Schmeisser et al. [93] reported that the administration of a 6-microbial phytase in a moderately phosphorous-deficient diet determined significant changes in the expression levels of genes involved in muscle development through calmodulin/calcineurin and insulin-like growth factor pathways. The activation of these pathways may have enhanced the development of breast muscle and increased its weight, even though no significant difference has been reported in terms of breast yield. Interestingly, birds received the dietary supplementation of dicalcium phosphate instead of phytase reported similar breast weight and yield compared to the phytase-supplemented group even though none of the previous pathways resulted significantly enriched. Therefore, the authors suggested that the muscle growth observed in these birds were not probably due to the same molecular mechanism [93]. The dietary administration of lysophospholipids-based emulsifiers has shown a positive effect on feed conversion rate in broiler chickens [94]. Microarray analysis performed on the jejunal epithelium of birds received the lysolecithin emulsifier showed an upregulation of genes for collagen, extracellular matrix, and integrins, suggesting that the positive effects of the emulsifier on productive performance might be achieved through changes in the intestinal epithelium [95]. Moreover, Khonyoung et al. [96] identified a higher expression of cluster of differentiation 36, an integral membrane protein involved in fat absorption, in jejunum of broilers fed diet supplemented with lysolecithin.

The European Union’s ban of antibiotics as growth promoters strengthened the interest towards alternative solutions that might provide beneficial effects on productive performance and health status of livestock. Prebiotics, such as yeast cell wall-derived compounds, are receiving even more attention due to their beneficial effects on growth performance, feed efficiency and gut health [97–99]. However, the molecular mechanism behind their effects has not been totally elucidated. Xiao et al. [100] applied a genome-wide transcriptional approach to investigate the effects of feeding mannan-oligosaccharides (MOS)-supplemented diets on jejunal gene expression of broiler chickens. Albeit they did not find any significant effect on productive performance, the transcriptomic analysis highlighted major expression of genes involved in protein synthesis, immune processes and antioxidant status in birds received MOS dietary supplementation. Moreover, several signaling pathways related to mitochondrial functions showed a potential involvement in mediating the effects of dietary MOS [100]. Furthermore, the beneficial effects of MOS have been associated with a reduced gut cell turnover and hence an increased energy preservation for growth, as indicated by the downregulation of genes involved in protein synthesis, protein metabolism, cellular assembly and organization, as well as the lower expression of genes of the mTOR pathway, in the intestinal mucosa of broilers receiving diets supplemented with MOS [101]. In addition, transcriptomic analysis evidenced common biological functions, such as antiviral and antimicrobial response, between birds receiving prebiotic or bacitracin supplemented diets, indicating that MOS may actively stimulate the intestinal innate immune system [101].

The dietary use of probiotics has been reported to be beneficial for chicken health and productivity [97, 102, 103]. Luo et al. [104] showed that the dietary supplementation of *Enterococcus faecium* had only a slightly positive effect on FCR, while stimulating the development of immune organs, number of intestinal microvilli and diversity of gut microflora. A proteomic approach carried out on the intestinal mucosa of the birds received the probiotic identified a total of 42 proteins showing differential expression, of which 60% could be associated with cytoskeleton and immune system. According to the authors, the probiotic may have enhanced FE through improving the absorptive area in the intestine while limiting the energy expenditure for immune system activation. It has been shown that the dietary supplementation of *E. faecium* can improve breast and legs yield, as well as water-holding capacity of meat, while determined low abdominal fat deposition [105]. The proteomic analysis performed on breast muscle allowed the identification of 22 differentially expressed proteins, mainly involved in carbohydrate and energy metabolism, as well as in cytoskeleton and molecular chaperones, which might have contributed to the improvements detected in carcass and meat quality. Recently, the dietary administration of *E. faecium* was associated with significant changes also in the liver proteome, indicating a potential effect in enhancing nutrient metabolism and partitioning as well as in decreasing the inflammatory response [106].

In the context of the microbiota, a huge number of published studies described the potential effect of dietary treatments in modifying (either successfully or not) the microbial consortium in the chicken gut (e.g. [107–109]). In addition to bacterial characterization, omics technologies can also be useful in understanding the metabolic functions performed by the bacteria communities colonizing the gut [34]. For example, the dietary administration of *Lactobacillus acidophilus* D2/CSL (CECT 4529) has been reported to improve body weight gain and feed efficiency, as well as the incidence of pasty vent in broiler chickens [110]. The DNA metagenomic sequencing analysis of cecum content identified significant changes in the microbiome of chickens fed or not the probiotic, even though the concentration of *Lactobacillus acidophilus* was similar between the groups. Considering metabolic functions, the bacterial communities of the supplemented group exhibited higher β-glucosidase levels, an enzyme involved in the hydrolysis of glucose monomers from non-starch polysaccharides and in the fermentation of undigested carbohydrates, which might have positively contributed to improving animal performance and gut health [110].

### Interaction between diet and animal genome

In order to improve important parameters such as feed efficiency and chicken meat quality, nutritionists must also consider the possible interaction between diet and animal’s genetic background. For instance, Jlali et al. [111] evaluated whether the polymorphisms identified in the promoter of *BCMO1* gene could affect the chicken response to the dietary β-carotene supplementation. To do that, homozygous chickens for *BCMO1* polymorphism (AA and GG) were fed a wheat-based diet supplemented or not with β-carotene. The results obtained in that study showed that the SNP in the promoter of *BCMO1* gene can determine tissue-specific changes in its expression and diet *x* genotype interactions for several physiological parameters. Indeed, in the GG genotype, the dietary administration of β-carotene increased the expression of intestine-specific homeobox (*ISX*) in duodenum, that in turn decreased the expression of *BCMO1*, suggesting a negative feedback mechanism able to maintain a steady level of retinol in the duodenum [111]. On the contrary, feeding AA birds with β-carotene supplemented diet did not have any significant effect on *BCMO1* and *ISX* levels in duodenum, and that might explain the observed accumulation of retinol in the duodenum. Therefore, these results showed a possible defect in the feedback regulation of duodenal *BCMO1* gene expression according to the bird genotype which may have marked implications in broiler nutrition. In another study, Jlali et al. [112] investigated the effects of feeding two isoenergetic diets with different crude protein levels on breast meat quality of two chicken lines divergently selected for abdominal fatness. Regardless of the chicken’s genotype, increasing the level of dietary protein has shown to be a nutritional strategy to improve body weight and breast yield but also to limit abdominal fatness. On the other hand, the reduction of dietary protein by 6% decreased breast muscle glycogen content, lightness and drip loss, while increased pHu value only in the lean line. At the molecular level, the lower glycogen content could be explained by the higher phosphorylation of the α-catalytic subunit of AMPK, which inhibits glycogen synthesis by phosphorylating the glycogen synthase [112]. Recently, Wen et al. [113] reported that low levels of dietary methionine negatively affect growth performance, carcass traits, meat quality attributes and oxidative status of breast muscle in a strain-dependent manner (fast- vs. slow-growing broiler chickens). However, no further investigation regarding the effect of the bird genotypes on the response to dietary methionine has been conducted in the study [113].

## Disease resistance

### Genetic resistance towards diseases

The poultry industry is strongly interested in preventing and controlling diseases to improve animal health, welfare, and productivity, as well as for maintaining consumer’s confidence and avoid trade restrictions. In chickens, beside the innate immunity that represents the first defensive line against invading pathogens, the adaptive immunity response operates through the communications of the antigen presenting cells, T and B cells, by direct contact with major histocompatibility complex (MHC), T cell receptor and immunoglobulins as well as secreted proteins such as cytokines and antibodies [114]. Individual differences in the immune response might be related to structural and functional variations of the above-mentioned molecules, which in turn can be attributed to intrinsic polymorphism of their encoding genes [114]. The application and integration of omics technologies could be useful to identify specific genes and genetic markers related to disease resistance in chickens [115]. Most of the genes located on chicken chromosome 16 showed a role in immune responses or at least appear to be involved in immunity by sequence homology with other species [116]. The MHC, or B complex, is a 242-kb region located on chromosome 16 and contains a broad number of genes, some of which are involved in resistance against to viral, bacterial, protozoal and autoimmune diseases [115, 116]. Most of the disease resistance associations are reported to be at the haplotype level, while the role of individual MHC genes in resistance to diseases was reported only in a few cases [116]. The role of MHC-B haplotypes in resistance against viral, bacterial and parasitic diseases was recently reviewed by Miller and Taylor [116]. Probably, the most important and well-characterized association of MHC with disease resistance is towards Marek’s disease (MD), a T-cell lymphoma of chickens caused by an oncogenic alpha-herpesvirus [114]. However, it has been reported that other genetic factors over than MHC genes might play an important role in resistance against Marek’s infection [115] and a total of 117 QTL was currently individuated for MD-related traits (http://www.animalgenome.org/cgi-bin/QTLdb/GG/index). An integration of genomic approaches to investigate MD resistance was reviewed by Cheng et al. [115]. Results from transcriptomic and pathway analysis indicated that chickens showing a more responsive immune system may be more susceptible to MD since the causative herpesvirus is thought to infect only activated lymphocytes [115]. Perumbakkam et al. [117] identified 6,132 SNP in 4,768 genes in broilers, as well as 4,528 SNP in 3,718 genes in layers, which exhibited allele-specific expression in response to MD virus infection. RNA-seq analysis identified 548 and 434 genes showing differential expression in broilers and layers after the infection, respectively. Even though broilers and layers showed substantial differences in enriched pathways after Marek virus infection, toll-like receptor and janus kinase/signal transducers and activators of transcription (JAK/STAT) signaling pathways were activated in both the genotypes when responding to the infection [117]. It has also been reported that 1,824 allele-specific expression SNP account for more than 83% of the genetic variance in resistance against MD in an experimental inbred layer population [118]. According to these results, the genetic merit of 200 rosters was predicted only using the allele-specific expression SNP and a progeny test was carried out. The progeny showed a reduction of 22% in the incidence of MD after only one generation of bidirectional selection based on those SNP. Moreover, the accuracy of the estimated breeding value was increased by 125% using the allele-specific expression SNP compared to the traditional pedigree-based method [118]. In order to better understand the genetic basis of the resistance against MD, other brilliant genome-wide [119, 120] and transcriptomic [121, 122] studies have been performed.

Salmonellosis represents one of the the main food-borne human diseases associated with the consumption of poultry products. According to the characteristics of different bacterial species, *Salmonella* might affect the birds at the systemic level (*Gallinarum* and *Pullorum* serovars) or causing a strong inflammatory response limited to the gastrointestinal tract (*Enteriditis* and *Thypimurium*) [115]. The latter case is the most dangerous for human health since Salmonella may establish a carrier state into the gastrointestinal tract of the chicken, becoming a potential source of contamination for poultry products [115]. Therefore, more resistant animals should be considered as those showing less intestinal colonization rather than those showing healthy conditions since most of them might appear asymptomatic to the infection [123]. The changes in the intestinal gene association networks in birds orally challenged with *Salmonella enteriditis* were described by Schokker et al. [124]. Calenge et al. [125] stated that the genomic regions containing genes as tool-like receptor 4 (*TLR4*) and natural resistance-associated macrophage protein [*NRAMP1*, now identified as solute carrier family 11 member 1 (*SLC11A1*)], as well as the MHC and the QTL SAL1, might be considered important candidates for controlling Salmonella infection in chickens. Overall, the other toll-like receptors, cytokines, antimicrobial β-defensins genes, as well as genes also related to T cell function and apoptosis, may play an important role [115]. Recently, other works seem to confirm the involvement of *TLR4* [126] and *NRAMP1* gene [127] in resistance against Salmonella. However, as observed by Calenge and Beaumont [123], none of the previous candidate genes showed a major and stable effect in several independent studies. Moreover, an epigenetic modification in leukocytes has been hypothesized to be linked to an increased susceptibility to *Salmonella enteritidis* infection by dampening the expression and the response of the different toll-like receptors [128]. A few years ago, Calenge et al. [129] reported a different genetic mechanism for controlling resistance to Salmonella carrier-state between animals of different age. Nonetheless, the authors observed a negative genetic correlation between chicks and hens resistance, indicating that increasing genetic resistance of hens could lead to a reduction of chicks’ resistance [129]. An overview of published study aimed at identifying genes and gene networks involved in the control of salmonellosis in chickens was recently published by Tohidi et al. [130].

Another important poultry disease is coccidiosis, which is an intestinal parasitosis caused by protozoans belonging to the genus *Eimeria* [131]. Overall, the host response to *Eimeria* infection is extremely complex determined by a broad range of biological processes which are controlled by many genes with a small effect and a limited number of genes with moderate or large effect [132]. Two important QTL involved in resistance to avian coccidiosis, LEI0071 [133] and LEI0101 [134], were identified on chicken chromosome 1. Moreover, the evaluation of the SNP in 3 candidate genes located between LEI0071 and LEI0101 identified *zyxin* as a potential candidate gene associated with resistance towards avian coccidiosis [135]. In a recent study conducted on broilers challenged with *Eimeria maxima*, significant associations have been identified between albumin levels, alpha 1 globulin, alpha 2 globulin, plasma coloration and regions on *Gallus gallus* chromosome 1, 2, and 6 [136]. Furthermore, genes and biological pathways involved in tissue repairment, general robustness, as well as primary immune response, may play a pivotal role during *Eimeria maxima* infection [136]. Previously, microarray analysis allowed the identification of at least 7 genes related to the interleukin signaling pathway showing differential expression in response to *E. maxima* infection [137]. The Authors also reported that the host response to the parasitic infection might involve a tightening of the epithelial barrier, enhancing the interaction between epithelial cells and the extracellular matrix through focal adhesions. Moreover, as indicated by the differential gene expression, it appeared that the innate immunity would be activated in the early phase of pathogen challenge, whereas the pattern of gene expression after a secondary infection indicated a rapid switch from an early innate response to a later adaptive one [137]. The use of capillary electrophoresis allowed to identify that total plasma protein content, as well as all fractions associated with acute phase proteins (mainly albumin, α1-globulin, α2-globulin, α3-globulin, β2-globulin), were significantly altered after a challenge with *Eimeria maxima* oocysts [132]. An innovative selection method based on the phenotypic expression of higher mRNA levels of pro-inflammatory mediators, such as cytokine and chemokine, allowed to improve resistance against *Salmonella enteritidis* [138], *Eimeria tenella* [139], *Clostridium perfringens*-induced necrotic enteritis [140] and *Campylobacter jejuni* ceca colonization [141]. Interestingly, while some QTL for *Salmonella* resistance resulted co-located with those for *Campylobacter* resistance [142], others are involved in antibody response for both *Salmonella* and *Escherichia coli* infection [143], paving the possibility to identify selection methods able to simultaneously increase resistance towards different pathogens [142]. Recently, the current knowledge regards the role of host immunity and genetic factors in necrotic enteritis was reviewed by Oh and Lillehoj [144].

### Immunomodulatory effects of nutrition

Nutrition can also affect the immune function of chickens [145] and appropriate dietary treatments can stimulate host immunity and influence their susceptibility toward diseases [146]. Omics technologies might be useful to evaluate wheatear a dietary treatment could be beneficial towards an inflammatory/pathological condition or not, and in the first case, which biological pathways and mechanisms are involved. Among amino acids, arginine appears to have an important effect on immune function and response. Dietary supplementation of *L*-arginine reduced intestinal mucosal disruption in coccidiosis-challenged chickens probably through suppressing TLR4 and activating mTOR complex 1 pathways [84]. Similarly, arginine supplementation attenuated systemic inflammation and reduced the overexpression of pro-inflammatory cytokines in chickens challenged with lipopolysaccharide mostly suppressing the TLR 4 pathway and CD14+ cell percentage in spleen [85]. Moreover, arginine exhibited a potential effect in alleviating the immunosuppression caused by infectious bursal disease vaccine through enhancing the immune function and modulating the circulating T cell sub-populations [86]. It has also been reported that threonine as well as methionine plus cysteine in-ovo administration can modulate the expression of immune genes in liver and intestine of broiler chickens [147]. Recently, the effects of dietary threonine, arginine and glutamine on intestinal mucosa health and integrity, as well as on the innate immune system activation, have been extensively reviewed by Bortoluzzi et al. [148].

It is widely known that some micronutrients, such as vitamins and minerals, may enhance humoral and cellular immune response. Considering the effect of vitamins, Gómez-Verduzco et al. [149] stated that high levels of vitamin D_3_ might be able to improve antibody and cellular immune response. Also dietary vitamin E could have an immunomodulatory effect particularly on CD4+/CD8- T cells in thymus and spleen [150] and might increase macrophage phagocytic activity during the early stages of broiler growth [151]. Selenium deficiency determined significant changes in selenoproteins expression, immunological alterations and oxidative stress in immune organs [152, 153], as well as changes in nitric oxide and heat shock proteins expression level in neutrophils [154]. On the other hand, selenium-enriched diets increased mRNA expression of selenoprotein W in thymus and in the bursa of Fabricius [155], in the gastrointestinal tract [156] and in liver [157]. In-ovo administration of selenium-containing protein hydrolysate enhanced protection against experimental necrotic enteritis likely through altering the expression of pro-inflammatory and antioxidant genes and their downstream pathways, as well as by enhancing immune and antioxidant response [158, 159]. Similarly, selenium dietary administration appears to be beneficial in reducing the detrimental effects of necrotic enteritis in young broilers [160].

Due to the European Union’s ban of antibiotics as growth promoters, there has been an increasing interest in evaluating the effects of the dietary administration of phytochemical compounds and plant extracts on health and immune status of the birds. It has been reported that extracts of *Curcuma longa* had a positive effect on coccidiosis resistance enhancing both systemic humoral and cellular immune response, as suggested by the differential expression of genes related to anti-inflammatory response in intestinal lymphocytes [161]. Similarly, two secondary metabolites of garlic, propyl thiosulphinate and propyl thiosulphinate oxide, showed a positive effect during *Eimeria acervulina* infection and the transcriptomic analysis of intestinal lymphocytes identified marked alterations in genes involved in immune pathways [162]. The intestinal lymphocytes transcriptomic profile of birds fed diets containing anethole and challenged with *Eimeria acervulina* showed “Inflammatory Response” as the most important function in the “Disease and Disorders” biological category, suggesting a beneficial effect of anethol against coccidiosis infection [163]. Furthermore, the effects of other phytonutrients in enhancing immune response in birds either challenged with experimental *Eimeria* infection [164] or not [165] have been reported. Lee et al. [166] showed that the dietary supplementation of *Allium hookeri* root or fermented root had a beneficial effect on gut health and inflammatory response in chickens stimulated with lipopolysaccharide through increasing the level of transcripts for tight junction proteins and mucin genes while reducing that of pro-inflammatory cytokines. Also the dietary administration of *Curcuma longa*, *Capsicum annuum*, *Capsicum frutescens* (hot pepper), and *Lentinus edodes* (shiitake mushroom) has shown to be beneficial towards avian coccidiosis as indicated by the higher serum antibody titers, as well as the increased transcripts level of interleukin 1-β, 6, and 15, and interferon γ, in duodenal mucosa [167]. Overall, the immune system appears as a very complicated and complex system. However, with an appropriate use of the new cutting-edge omics technologies we can shed some light and try to understand this complexity, allowing for the future the selection of more resistant birds as well as understanding the potential role of nutrients and feed additives as immunomodulatory compounds.

## Conclusions

The application of omics technologies allowed extraordinary progresses in studying and understanding many quali-quantitative traits in broilers. In particular, transcriptomics, proteomics and metabolomics approaches have been successfully applied for investigating the molecular basis of complex traits such as feed efficiency and muscle myopathies, as well as for assessing the molecular responses to nutritional treatments and for evaluating important aspects related to immunity and disease resistance. However, there is still room to improve the knowledge regarding the molecular aspects involved in different traits of avian species. First of all, most of the published studies speculate about sets of differentially expressed genes and proteins during specific conditions. Anyhow, we should evaluate whether the up- or down-regulation of specific gene products could be considered as the determinant of the phenotypic expression of a specific trait or it might be a molecular “response” to that condition. Therefore, many more mechanistic studies are necessary to understand in detail several molecular aspects behind quali-quantitative traits of broiler chickens. Moreover, most of the current studies are mainly focused on vital economic traits for the poultry industry, such as feed efficiency, breast meat yield and meat quality. However, the application of omics technologies might be useful for investigating and try to find out solutions to the new challenges that the poultry industry is currently facing (e.g. sex determination prior to hatch, response to heat stress conditions, welfare and sustainability issues and antimicrobial resistance). Moreover, from a genetic point of view, there are many other genic products, such as non-coding RNA, micro-RNA and short interfering RNA, which deserve much more attention as their role in many important biological processes of the chickens has been established but not fully understood yet.

## Abbreviations

25(OH)D_3_: 25-Hydroxycholecalciferol
3HADH: 3-Hydroxyacyl-CoA dehydrogenase
AMPK: Adenosine monophosphate AMP-activated protein kinase
avANT: Avian adenine nucleotide translocator
avTOR: Avian target of rapamycin
avUPC: Avian uncoupling protein
BCMO1: Beta-carotene monooxygenase 1
COX III: Cytochrome oxidase III
CPT1: Carnitine palmitoyl transferase1
eIF2: Eukaryotic initiation factor 2
eIF4: Eukaryotic initiation factor 4
ETC: Electron transport chain
FAS: Fatty acid synthase
FCR: Feed conversion rate
FE: Feed efficiency
IGFs/PI3K/Akt: Insulin-like growth factor-I/phosphatidylinositol 3-kinase/protein kinase B
iNOS: Inducible nitric oxide synthase
ISX: Intestine-specific homeobox
JAK/STAT: Janus kinase/signal transducers and activators of transcription
Jnk: c-Jun NH(2)-terminal protein kinase
MAP4K4: Mitogen-activated protein kinase 4
MD: Marek’s disease
MHC: Major histocompatibility complex
MOS: Mannan oligosaccharide
MS: Mass spectrometry
mTOR: Mechanistic target of rapamycin
NMR: Nuclear magnetic resonance
NRAMP1: Natural resistance-associated macrophage protein
NSRT-PCR: Real-time quantitative reverse transcription PCR
p70S6K: 70 kDa ribosomal S6 kinase
PGC-1α: PPAR-γ coactivator-1α
pHu: Ultimate pH
PKA: Protein kinase-A
PPAR-γ: Peroxisome proliferator-activated receptor-γ
PRKAγ2: Protein kinase AMP-activated non-catalytic subunit gamma 2
QTL: Quantitative Trait Loci
RAR-RXR: Retinoic acid and retinoid X receptor
RFI: Residual feed intake
RICTOR: Rapamycin independent companion of target of rapamycin
RNA-seq: RNA-sequencing
ROS: Reactive oxygen species
SM: Spaghetti meat
SNP: Single nucleotide polymorphisms
TLR4: Toll-like receptor 4
WB: Wooden breast
WS: White striping
